# Supplementary material for: The impact of brain iron accumulation on cognition: A systematic review
Source: PLoS One. 2020 Oct 15;15(10):e0240697. doi: 10.1371/journal.pone.0240697 (PMC7561208; doi:10.1371/journal.pone.0240697)
Supplement: Attachment — Submitted filename: Notes PONE-D-20-20440.docx [file pone.0240697.s002.docx]

**#Referee**

Comments to the Corresponding Author

PONE-D-20-20440

The authors have invested in a systematic approach to compare and evaluate the current literature and explore the relationship between brain iron and cognition. Identifying that iron appears to accumulate heterogeneously in regions of the brain and when only a few regions have a high iron content, its specific function may be deficient. Thus, increase iron in the caudate nuclei, hippocampus and thalamus is correlated with lower memory performance; whereas, increase iron in the putamen has been reported more consistently to correlate with poorer general cognition. The data presented here is very interesting, and the study design was generally successful.

However, the following weaknesses and improvements must be considered:

Point 1. However, according to Bartzokis et al. (2007) ferritin iron levels are higher in men than in women, which may contribute to the risk of developing neurodegenerative diseases at an early age. In this review, the gender factor was not discussed, according to the studies consulted, there would be changes in iron levels in women? And what would be the average age of both sexes attributed to cognitive impairment due to high levels of iron?

Point 2. It is suggested that there is more work /reflection on how high levels of iron act on the nervous system and cause cognitive alterations.

Line-By-Line

Point 3. Line 41- “In the healthy adult brain”. Please specify whether the quote refers to humans?

Point 4. Line 54- define ROS before using the acronym.

Point 5. Line 56- What damage excessive ROS generation can cause at the brain level, how neurodegeneration can occur via Fenton Reaction?

Point 6. Line 84- PD e AD previously abbreviated.

Point 7. Line 89- The systematic review brings gaps to be answered and hypotheses to be raised, the addition of a hypothesis to the study is encouraged.

Point 8. Line 115- define HS before using the acronym.

Point 9. Line 142- In this sentence it indicates that 43 studies were used, however, in Table 2 and Table 3 there is a citation of 41 studies.

Point 10. Line 143- Figure 1 is somewhat unreadable.

Point 11. Line 149 and 157- Standardize the use of abbreviations in tables.

Point 12. Line 126-127- Previously abbreviated.

Point 13. Line 237- define Aβ before using the acronym.

Point 14. Line 253-“all included studies were published articles or theses”. It is necessary to insert in the material and methods the use of theses in this review.

Point 15. Line 265-267- The insertion of a graphic scheme is strongly encouraged, making these possible correlations.
